# Why be original? Two new species of *Choeradoplana* resembling the type species of the genus in their external aspects (Platyhelminthes, Continenticola)

**DOI:** 10.3897/zookeys.813.29565

**Published:** 2019-01-07

**Authors:** Giuly Gouvêa turralde, Ana Leal-Zanchet

**Affiliations:** 1 Instituto de Pesquisas de Planárias and Programa de Pós-Graduação em Biologia, Universidade do Vale do Rio dos Sinos, 93022-750 São Leopoldo, Rio Grande do Sul, Brazil Universidade do Vale do Rio dos Sinos São Leopoldo Brazil

**Keywords:** *Araucaria* Forest, Neotropical region, taxonomy, Tricladida

## Abstract

The genus *Choeradoplana* Graff, 1896 encompasses 16 species, most of them found in Brazil. Herein two new species of this genus are described from remnants of *Araucaria* moist forests, located in the states of Paraná and Santa Catarina, south Brazil. Both species resemble the type-species of the genus, *C.iheringi*, showing brownish dorsal surface covered by dark-brown flecks. However, regarding their anatomy, the new species differ from *C.iheringi* and other congeners by a long and horizontal disposed permanent papilla. In such aspects, both species resemble *C.benyai*, but differ from this species, as well as from each other, in details of the prostatic vesicle, ejaculatory duct, and penis papilla.

## Introduction

The genus *Choeradoplana*, proposed by Graff (1896), has a Neotropical distribution, with most species recorded from Brazil. Its representatives show a cephalic region with a glandulo-muscular organ and longitudinal cutaneous musculature with a portion sunk into the mesenchyme (Graff 1899, Ogren and Kawakatsu 1990). The genus was reviewed by Froehlich (1955). Carbayo and Leal-Zanchet (2003) and Carbayo and Froehlich (2012) suggested the inclusion of other features in its diagnosis. Currently, the genus encompasses 16 species, six of them occurring in areas of ombrophilous *Araucaria* forest (Graff 1899, Ogren and Kawakatsu 1990, Leal-Zanchet and Souza 2003, Carbayo and Froehlich 2012, Negrete and Brusa 2012, Lemos et al. 2014, Álvarez-Presas et al. 2017).

The *Araucaria* forest is a phytophysiognomy of the Atlantic Forest, which harbours a high species richness of land flatworms (Sluys 1998, Leal-Zanchet and Carbayo 2000, Castro and Leal-Zanchet 2005, Antunes et al. 2008, Leal-Zanchet and Baptista 2009; Baptista et al. 2010, Leal-Zanchet et al. 2011, Amaral et al. 2014). A recent inventory of land flatworms in areas of *Araucaria* moist forest located in the states of Paraná and Santa Catarina, in south Brazil, indicated the occurrence of brownish specimens with dark-brown flecks over the dorsal surface, similar to the type-species of the genus, *Choeradoplanaiheringi* Graff, 1899. Anatomical and histological analyses indicated that they belong to two different species that are herein described.

## Materials and methods

Flatworms were sampled in two protected areas located in the Iguassu River Drainage Basin, in south Brazil, namely the *Araucaria* Natural Heritage Private Reserve (26°20.35'–26°26.13'S; 51°19.49'–51°25.29'W), in General Carneiro, state of Paraná, and Três Barras National Forest (26°09.27'–26°16.9'S; 50°16.0'–50°21.22'W), in Três Barras, state of Santa Catarina. Specimens were collected by visual search during the night, when they are more active.

Just after sampling, colour pattern, body shape and dimensions of live specimens were recorded. They were then euthanised using boiling water and fixed in neutral formalin 10%. After fixation, specimens were maintained in 70% ethyl alcohol. Methods described by Rossi et al. (2015) were used for histological processing of material and analysis of external and internal characteristics. The material was sectioned at intervals of 6 µm and stained with Masson’s trichrome method or Haematoxylin and Eosin (Romeis 1989).

Type-material is deposited in the Museu de Zoologia da Universidade do Vale do Rio dos Sinos, São Leopoldo, state of Rio Grande do Sul, Brazil (MZU), and the Helminthological Collection of Museu de Zoologia da Universidade de São Paulo, state of São Paulo, Brazil (**MZUSP**).

Abbreviations used in the figures:

**cmc** common muscle coat;

**cov** common glandular ovovitelline duct;

**cs** creeping sole

**de** dorsal epidermis;

**di** dorsal insertion of pharynx;

**dm** dorsal cutaneous musculature;

**e** eyes;

**ej** ejaculatory duct;

**fc** female canal;

**gc** glandular cushions;

**go** gonopore;

**i** intestine;

**im** internal musculature;

**m** mouth;

**mm** mesenchymal muscles;

**mn** “muscle net”;

**n** nerve plate;

**nlm** normal longitudinal cutaneous muscles;

**om** outer musculature;

**ov** ovovitelline ducts;

**p** penis papilla;

**pp** pharyngeal pouch;

**pv** prostatic vesicle;

**r** retractor muscle;

**rg** rhabditogen glands;

**sc** secretory cells;

**sg** shell glands;

**slm** sunken longitudinal cutaneous muscles;

**sv** spermiducal vesicle;

**t** testes;

**v** vitelline follicles;

**ve** ventral epidermis;

**vi** ventral insertion of pharynx.

## Taxonomy

### Family Geoplanidae Stimpson, 1857

#### Subfamily Geoplaninae Stimpson, 1857

##### Genus *Choeradoplana* Graff, 1896

###### Choeradoplana longivesicula sp. n.

Taxon classificationAnimaliaTricladidaGeoplanidae

http://zoobank.org/FC60603B-DBA9-4B55-BB17-FF9E0B337C61

Figures 1–2 , 3–7 , 8, 9 , 10–11

####### Type-material.

**Holotype**: MZUSP PL.2143: leg. JAL Braccini, 6 June 2015, General Carneiro (*Araucaria* Natural Heritage Private Reserve), PR, Brazil – anterior tip: transverse sections on 24 slides; anterior region at the level of the ovaries: sagittal sections on 27 slides; pre-pharyngeal region: transverse sections on 8 slides; pharynx: sagittal sections on 31 slides; copulatory apparatus: sagittal sections on 25 slides. **Paratype**: MZU PL.00292: leg. I Rossi, 6 June 2015, General Carneiro (*Araucaria* Natural Heritage Private Reserve), state of Parana, Brazil – anterior tip: transverse sections on 27 slides; anterior region at the level of the ovaries: sagittal sections on 24 slides; pre-pharyngeal region: transverse sections on 10 slides; pharynx: sagittal sections on 22 slides; copulatory apparatus: sagittal sections on 19 slides.

####### Diagnosis.

A species of *Choeradoplana* with dorsal surface covered by irregular small dark-brown flecks and thin median light stripe; pharynx bell-shaped; sperm ducts opening laterally into proximal wall of prostatic vesicle; prostatic vesicle tubular and unpaired, narrowing to open through tip of penis papilla as an ejaculatory duct; penis papilla cylindrical and almost symmetrical, filling entire common atrium; atrium oval-elongate and unfolded, without differentiation between male and female regions.

####### Description.

**Figures 1, 2.**
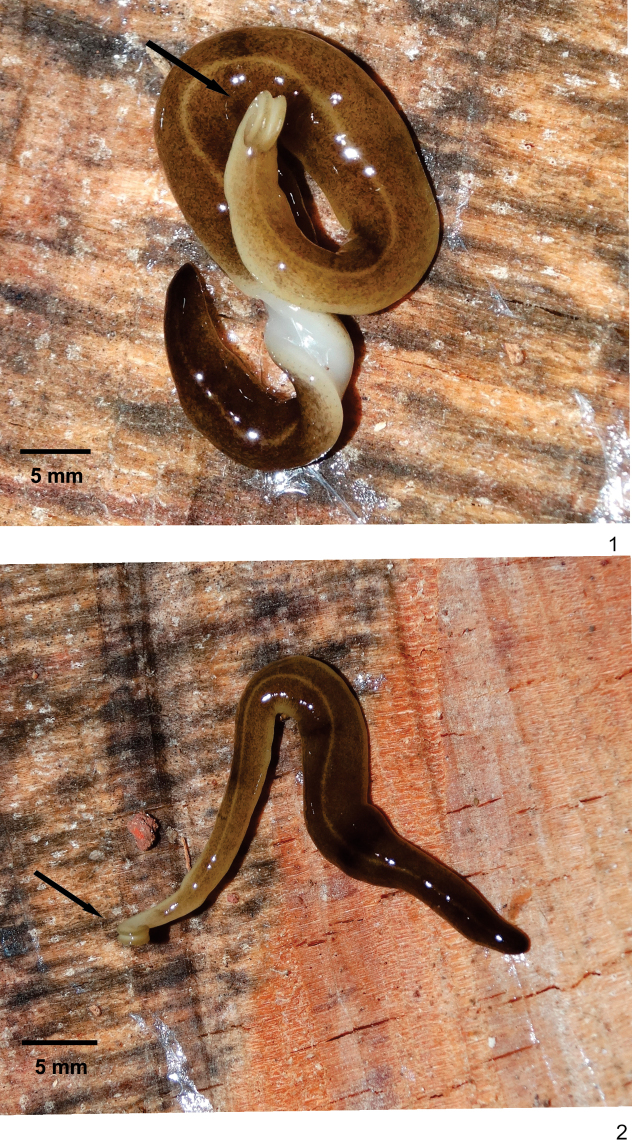
*Choeradoplanalongivesicula* sp. n., paratype MZU PL. 00292, **1, 2** dorsal view, with part of whitish ventral surface visible in **1**. Arrows indicate the anterior tip.

**External features.** Body elongate with parallel margins (Figs 1, 2), elliptical in cross section; anterior end expanded, posterior end slight pointed. Cephalic region (ca. 3 mm long) with two glandular cushions separated by a median longitudinal slit in the ventral surface. Maximum length 67 mm when crawling; 50 mm after fixation (Table 1). Mouth at median third of body; gonopore at posterior third of body (Table 1).

**Table 1.** Measurements, in mm, of specimens of *Choeradoplanalongivesicula* sp. n. Abbreviations: * after fixation; DG distance of gonopore from anterior end; DM distance of mouth from anterior end; DMG distance between mouth and gonopore; DPVP distance between prostatic vesicle and pharyngeal pouch. The numbers given in parentheses represent the position relative to body length.

Measurement	Holotype MZUSP PL. 2143	Paratype MZU PL.00292
Length at rest	34	47
Width at rest	6	4
Maximum length in extension	62	67
Maximum width in extension	3	3
Length*	52	49
Width*	4	4
DM*	33(63)	30 (61)
DG*	39(75)	35 (71)
DMG*	6	5
DPVP*	1.8	1.6
Creeping sole %	86	82
Ovaries	18(35)	16 (32)
Anteriormost testes	17(33)	15 (30)
Posteriormost testes	28(54)	26 (53)
Length of prostatic vesicle	2.5	2.3
Length of penis papilla	2.1	1.9
Length of atrium	2.4	2.3
Female canal	0.8	0.7
Common glandular ovovitelline duct	0.1	0.1

Live specimens with dorsal surface covered by dark-brown pigmentation constituted by irregular, small flecks. Yellowish ground colour visible on cephalic region, on body margins, as well as on thin median stripe occurring along the body except for cephalic region (Figs 1, 2). Ventral surface whitish before and after fixation. After fixation, dorsal pigmentation fades.

Eyes absent on anterior tip (first 1.5 mm of body, corresponding to 3% of body length). Afterwards, eyes initially monolobate and uniserial. Eyes become trilobate and plurisserial after 3 mm and sparser towards posterior tip. No clear halos around eyes. Pigment cups between 20 µm and 30 µm in diameter.

**Figures 3–7.**
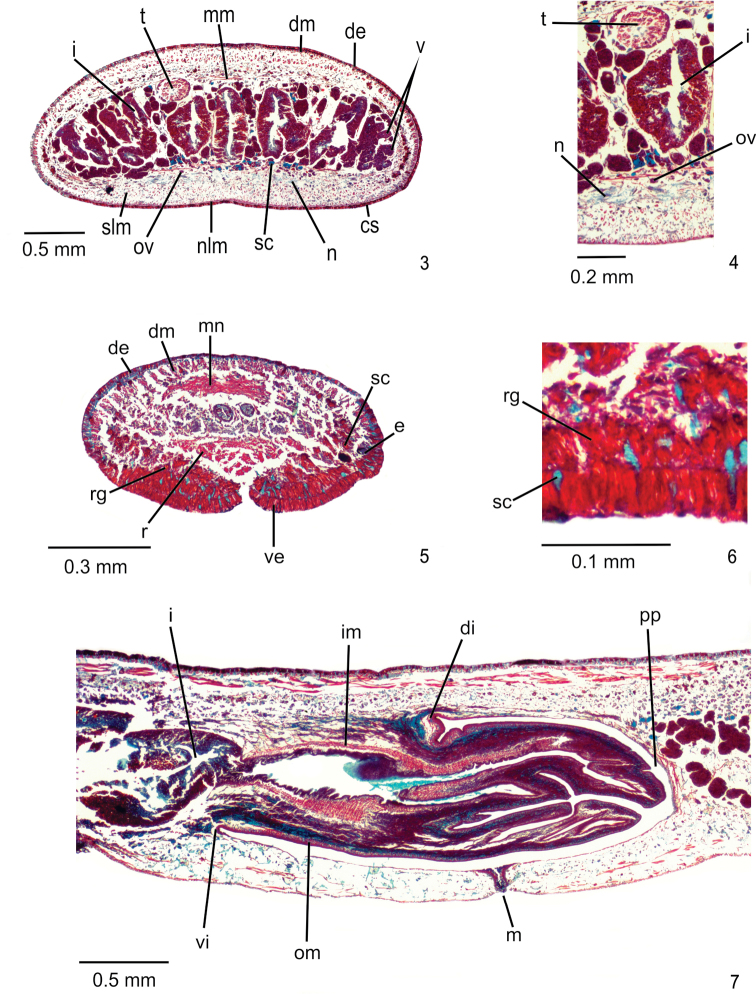
*Choeradoplanalongivesicula* sp. n., paratype MZU PL. 00292 **3, 4** pre-pharyngeal region, transverse sections **5, 6** anterior region of body, transverse sections **7** pharynx, sagittal section, with anterior tip to the left.

**Sensory organs, epidermis and body musculature.** Sensory pits, as simple invaginations (15–20 µm deep), absent on anterior tip, occurring in a single row between 3% and 10% of body length. Three types of glands discharge through whole epidermis of pre-pharyngeal region: rhabditogen glands with xanthophil rhammites (ventrally with smaller rhabdites) and cyanophil glands with amorphous secretion, as well as few erythrophil glands with fine granular secretion (Figs 3, 4). Creeping sole occupies 82% of body width. Glandular margin absent. Glands discharging through anterior tip of body with similar arrangement as in other species of the genus.

Cutaneous musculature with usual three layers (circular, oblique, and longitudinal layers), showing part of ventral longitudinal layer, as well as some muscle bundles of dorsal longitudinal layer, imbedded in mesenchyme (Fig. 3, Table 2). Longitudinal layer between four and eight times thicker than other two cutaneous layers in pre-pharyngeal region (Table 2). Cutaneous musculature as thick paramedially as medially. Ventral musculature slightly thinner than dorsal in pre-pharyngeal region. Ratio between cutaneous musculature and body height (mc:h) ca. 19% (Table 2). In cephalic region, cutaneous musculature with similar arrangement as in other species of the genus (Fig. 5).

**Table 2.** Body height and cutaneous musculature in the median region of a transverse section of the pre-pharyngeal, in micrometres, and ratio of the thickness of cutaneous musculature to the height of the body (mc:h index) of specimens of *Choeradoplanalongivesicula* sp. n.

Measurement	Holotype MZUSP PL.2143	Paratype MZU PL.00292
Dorsal circular	3	4
Dorsal oblique	25	24
Dorsal longitudinal	100	108
Dorsal imbedded	20	18
Dorsal total	148	154
Ventral circular	2	3
Ventral oblique	15	13
Ventral longitudinal	40	38
Ventral imbedded	70	67
Ventral total	127	121
Body height	1421	1413
Mc:h(%)	19	19

Mesenchymal musculature (Fig. 3) weakly developed, mainly composed of three layers: (1) dorsal subcutaneous, located mainly close to cutaneous musculature, with decussate fibres (2–3 fibres thick), (2) supra-intestinal transverse (3–5 fibres thick) and (3) sub-intestinal transverse (4–6 fibres thick). In cephalic region, mesenchymal musculature with similar arrangement as in other species of the genus (Fig. 5).

**Digestive System.** Pharynx bell-shaped, ca. 6% of body length, occupies ca. 90% of pharyngeal pouch. Mouth slightly posterior to dorsal insertion next to end of median third of pharyngeal pouch (Fig. 7). Oesophagus absent.

**Figures 8, 9.**
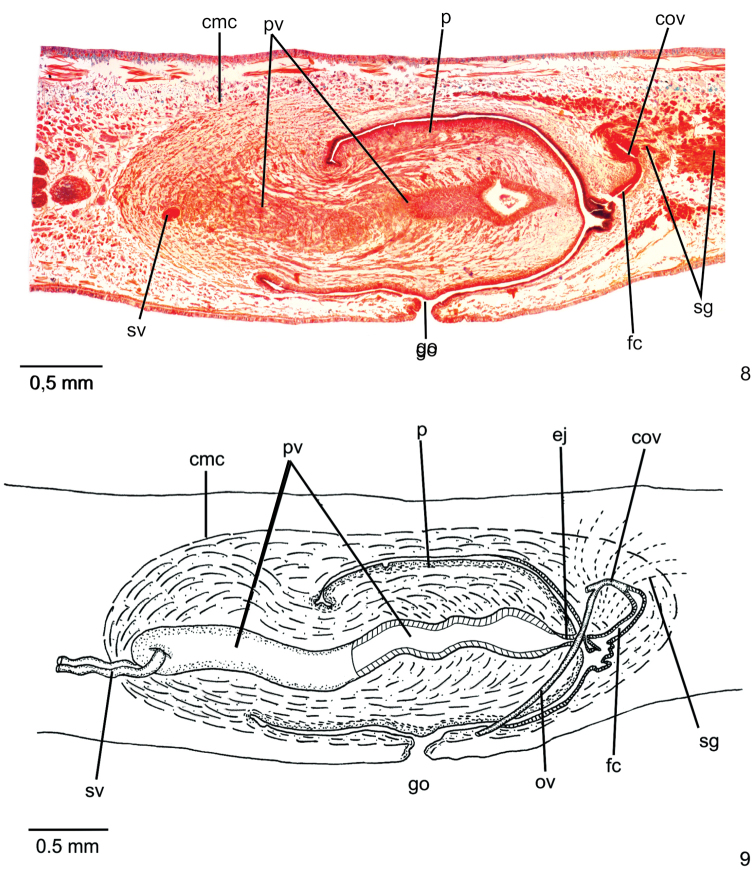
*Choeradoplanalongivesicula* sp. n., holotype **8** copulatory apparatus, sagittal section **9** sagittal composite reconstruction of copulatory apparatus. Anterior tip to the left.

**Figures 10, 11.**
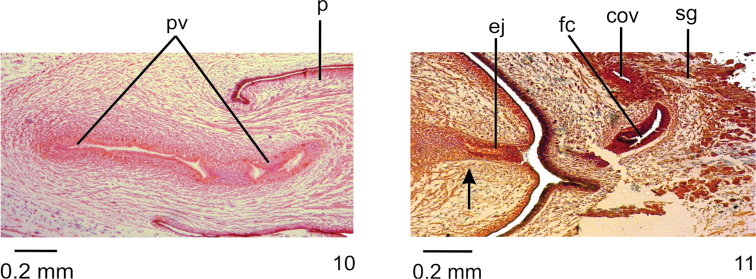
*Choeradoplanalongivesicula* sp. n., holotype, sagittal sections **10** prostatic vesicle **11** ejaculatory duct and female canal. Anterior tip to the left. Arrow indicates the transition from the prostatic vesicle to the ejaculatory duct.

**Reproductive organs**. Testes in one or two irregular rows on either side of body, located beneath dorsal transverse mesenchymal muscles, between intestinal branches (Fig. 3), begin slightly anteriorly to ovaries, in anterior third of body, and extend to next to root of the pharynx (Table 1). Sperm ducts dorsal to ovovitelline ducts, under or among fibres of sub-intestinal transverse mesenchymal musculature, in pre-pharyngeal region (Figure 3). They form spermiducal vesicles posteriorly to pharynx. Sperm ducts enter common muscle coat, ascend slightly and open laterally into proximal wall of prostatic vesicle. Intrabulbar prostatic vesicle, tubular and unpaired, traverses both penis bulb and papilla (Fig. 10), narrowing to open through tip of papilla as an ejaculatory duct (Fig. 11). Penis papilla, cylindrical and almost symmetrical, filling entire common atrium. Dorsal insertion of penis papilla slightly shifted posteriorly. Common atrium oval-elongate and unfolded, without differentiation between male and female atria (Figs 8, 9).

Prostatic vesicle lined with high pseudostratified epithelium with few ciliated cells, receiving numerous openings of glands with ill-stained, coarse granular secretion, sometimes containing an erythrophil core. In addition, sparse openings of other two types of glands occur throughout the epithelium of prostatic vesicle: cells with xanthophil, coarse granular secretion, and cell with amorphous, cyanophil secretion. Ejaculatory duct lined with columnar, densely ciliated epithelium. Both penis papilla and atrium lined with non-ciliated columnar epithelium, becoming cuboidal towards tip of papilla, showing a xanthophil apical region. Numerous glands of two types open through epithelial lining of penis papilla and atrium: one with xanthophil, coarse granular secretion and the other with amorphous, cyanophil secretion. In addition, a third type, with fine granular, erythrophil secretion, opens through epithelial lining of penis papilla. Muscularis of penis papilla (30–60 µm) composed of subepithelial layer with circular fibres followed by some longitudinal fibres; that of atrium composed of longitudinal and circular interwoven fibres (10–15 µm).

Vitelline follicles situated between intestinal branches, well developed. Ovaries ovoid, ca. twice longer than wide, measuring 0.2 mm in its antero-posterior axis. They are located dorsally to the ventral nerve plate, in anterior third of body. Ovovitelline ducts emerge laterally from median third of ovaries, then run posteriorly immediately dorsal to the nerve plate. Behind the gonopore, the ovovitelline ducts ascend posteriorly and medially inclined, and unite, dorsally to the female canal, forming the common glandular ovovitelline duct. Female canal relatively long and C shaped. This canal opens into bottom of posterior part of atrium, where a constriction occurs (Figs 8, 9).

Female canal lined with erythrophil, pseudostratified epithelium. Three types of glands open through the epithelium of female canal: abundant cells with finely granular, erythrophil secretion, cells with coarse granular, xanthophil secretion, and scarce cells with amorphous, cyanophil secretion. Muscularis of female canal composed of longitudinal and circular interwoven fibres (20–30 µm)

Gonopore canal almost vertical at the sagittal plane. Common muscle coat highly developed, especially at penis bulb (Figs 8, 9), with interwoven oblique, circular and longitudinal fibres.

####### Etymology.

The name is composed of the Latin adjective *longus* (long) and the Latin *vesicula*, alluding to the elongate prostatic vesicle.

####### Distribution.

known only from the type-locality, General Carneiro, Paraná, Brazil.

###### Choeradoplana cyanoatria sp. n.

Taxon classificationAnimaliaTricladidaGeoplanidae

http://zoobank.org/424F7AF0-33EE-4ED0-9500-27EB77503752

**Figure 12.**
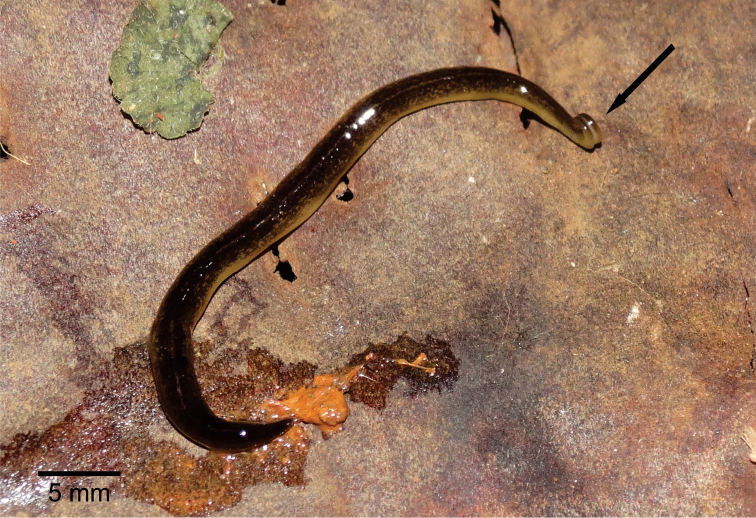
*Choeradoplanacyanoatria* sp. n., holotype, dorsal view. Arrow indicates the anterior tip.

**Figures 13, 14.**
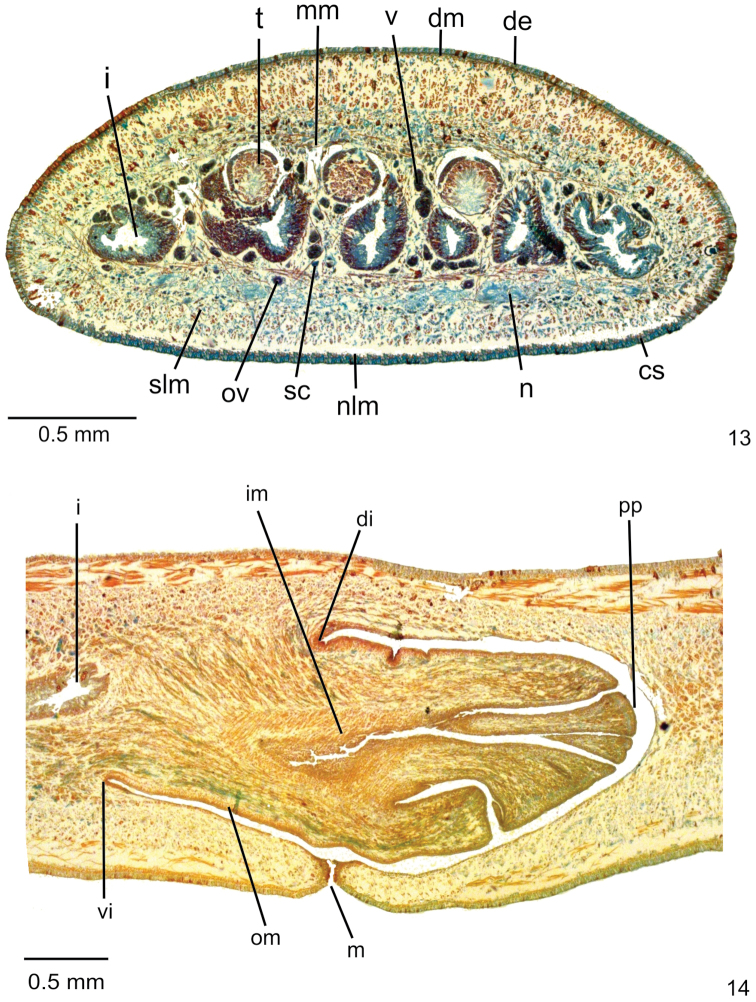
*Choeradoplanacyanoatria* sp. n., holotype **13** pre-pharyngeal region, transverse section **14** pharynx, sagittal section, with anterior tip to the left.

**Figures 15, 16.**
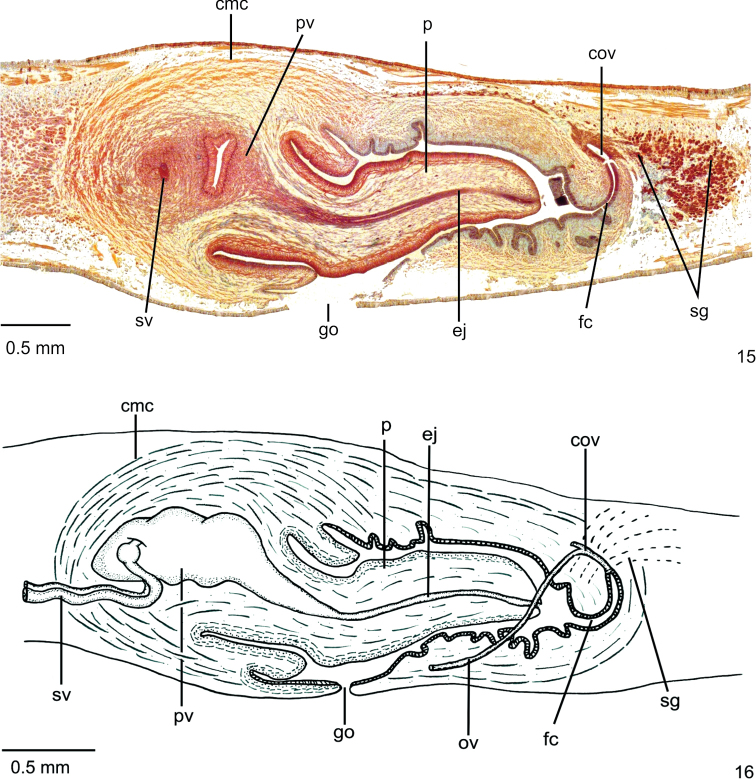
*Choeradoplanacyanoatria* sp. n., holotype **15** copulatory apparatus, sagittal section **16** sagittal composite reconstruction of copulatory apparatus. Anterior tip to the left.

**Figures 17, 18.**
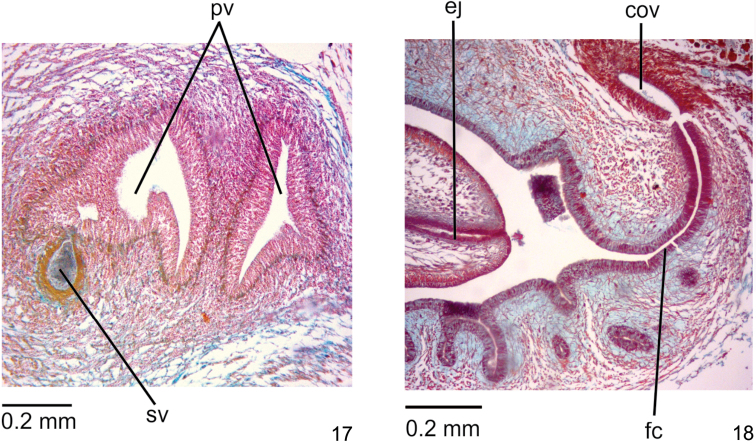
*Choeradoplanacyanoatria* sp. n., holotype, sagittal sections **17** prostatic vesicle **18** ejaculatory duct, and female canal. Anterior tip to the left.

Figures 12 , 13, 14 , 15, 16 , 17–18

####### Type-material.

**Holotype**: MZUSP PL.2144: leg. JAL Braccini, 2 June 2015, Três Barras (National Forest), state of Santa Catarina, Brazil – anterior tip: transverse sections on 17 slides; anterior region at the level of the ovaries: sagittal sections on 16 slides; pre-pharyngeal region: transverse sections on 7 slides; pharynx and copulatory apparatus: sagittal sections on 25 slides.

####### Diagnosis.

A species of *Choeradoplana* with dorsal surface covered by irregular small dark-brown flecks; pharynx bell-shaped; sperm ducts opening subterminally into prostatic vesicle; prostatic vesicle oval-elongate and folded, becoming funnel-shaped proximally and forming an elongate duct inside penis papilla; penis papilla, conical, long and almost symmetrical, with dorsal insertion shifted posteriorly, filling the whole atrium.

####### Description.

**External features.** Body elongate with parallel margins (Fig. 12), sub-cylindrical in cross section; anterior end expanded, posterior end slight pointed. Cephalic region (ca. 3 mm long) with two glandular cushions and a median slit in the ventral surface. Maximum length 20 mm when resting; 50 mm after fixation (Table 3). Mouth at median third of body; gonopore at posterior third of body (Table 3).

**Table 3.** Measurements, in mm, of the holotype of *Choeradoplanacyanoatria* sp. n. Abbreviations: * after fixation; DG distance of gonopore from anterior end; DM distance of mouth from anterior end; DMG distance between mouth and gonopore; DPVP distance between prostatic vesicle and pharyngeal pouch. The numbers given in parentheses represent the position relative to body length.

Measurement	HolotypeMZUSP PL.2144
Length at rest	20
Width at rest	4
Maximum length in extension	28
Maximum width in extension	3
Length*	50
Width*	4
DM*	33(66)
DG*	40(80)
DMG*	7
DPVP*	1.2
Creeping sole %	89
Ovaries	8 (16)
Anteriormost testes	9 (18)
Posteriormost testes	29 (58)
Length of prostatic vesicle	0.6
Length of penis papilla	1.3
Length of the atrium	1.6
Female canal	0.4
Common glandular ovovitelline duct	0.1

Live specimens with dorsal surface covered by irregular, small dark-brown flecks over all body length including cephalic region (Fig. 12). Yellowish ground colour visible on cephalic region, on body margins, as well as on thin, inconspicuous median stripe occurring along the anterior body half except for cephalic region. Ventral surface pale yellow. After fixation, dorsal pigmentation remains brownish; ventral surface becomes whitish with darker body margins.

Eyes absent on cephalic region (first 1.2 mm of body, corresponding to 2.4% of body length). After that, eyes initially monolobate and uniserial. Eyes become trilobate and plurisserial after 3 mm, becoming sparser towards posterior tip. No clear halos around eyes. Diameter of pigment cups between 24 µm and 32 µm in diameter.

**Sensory organs, epidermis and body musculature**. Sensory pits, as simple invaginations (15–18 µm deep), absent on anterior tip, occurring in a single row between 3% and 10% of body length.

Three types of glands discharge through whole epidermis of pre-pharyngeal region: rhabditogen glands with xanthophil rhammites (ventrally with smaller rhabdites) and cyanophil glands with amorphous secretion, as well as few xanthophil glands with coarse granular secretion (Fig. 13). Creeping sole occupies 89% of body width. Glandular margin absent. Glands discharging through anterior tip of body with similar arrangement as in other species of the genus.

Cutaneous musculature with usual three layers (circular, oblique, and longitudinal layers), with part of ventral longitudinal layer, as well as few muscle bundles of dorsal longitudinal layer, imbedded in mesenchyme (Fig. 13, Table 4). Longitudinal layer between five and eight times thicker than other two cutaneous layers in pre-pharyngeal region (Table 4). Cutaneous musculature as high paramedially as medially. Ventral musculature thinner than dorsal in pre-pharyngeal region. Mc:h 22% (Table 4). In cephalic region, cutaneous musculature with similar arrangement as in other species of the genus.

**Table 4.** Body height and cutaneous musculature in the median region of a transverse section of the pre-pharyngeal, in micrometres, and ratio of the thickness of cutaneous musculature to the height of the body (mc:h index) of the holotype of *Choeradoplanacyanoatria*.

Measurement	Holotype MZUSP PL.2144
Dorsal circular	4
Dorsal oblique	8
Dorsal longitudinal	33
Dorsal imbedded	27
Dorsal total	72
Ventral circular	2
Ventral oblique	15
Ventral longitudinal	130
Ventral imbedded	5
Ventral total	152
Body height	1025
Mc:h(%)	22

Mesenchymal musculature (Fig. 13) weakly developed, mainly composed of three layers: (1) dorsal subcutaneous, located mainly close to cutaneous musculature, with decussate fibres (2 fibres thick), (2) supra-intestinal transverse (2–4 fibres thick) and (3) sub-intestinal transverse (5–7 fibres thick). In cephalic region, mesenchymal musculature with similar arrangement as in other species of the genus.

**Digestive system.** Pharynx bell-shaped, as long as 7% of body length, occupies almost entire pharyngeal pouch. Mouth almost at the same transversal level as dorsal insertion in the beginning of median third of pharyngeal pouch (Fig. 14). Oesophagus absent.

**Reproductive organs.** Testes in two or three irregular rows on either side of body, located beneath dorsal transverse mesenchymal muscles, between intestinal branches (Fig. 13). They begin slightly anteriorly to ovaries, in anterior sixth of body, to just the root of the pharynx (Table 3). Sperm ducts dorsal to ovovitelline ducts, medially displaced, under or among fibres of sub-intestinal transverse mesenchymal musculature, in pre-pharyngeal region (Fig. 13). They form spermiducal vesicles posteriorly to pharynx. Sperm ducts enter common muscle coat, recurve, and open subterminally into prostatic vesicle. Intrabulbar prostatic vesicle, oval-elongate and folded, becoming funnel-shaped both proximally and distally (Fig. 17). Inside penis papilla, prostatic vesicle narrows and forms an elongate duct that opens through tip of the papilla. Penis papilla, conical, long and almost symmetrical, filling the whole atrium. The dorsal insertion of the penis papilla is posteriorly shifted (Figs 15, 16). Folded atrium without anatomical or histological differentiation between male and female regions. Close to papilla insertions, longitudinal folds represent part of papilla wall.

Prostatic vesicle and proximal third of ejaculatory duct receive abundant openings of cells with coarse granular, erythrophil secretion, besides sparse amorphous, cyanophil secretion, besides a third type of gland containing heavy cyanophil granules. Distal two thirds of ejaculatory duct receives openings from numerous glands with amorphous, cyanophil secretion. Muscularis of ejaculatory duct thin (5µm) composed of longitudinal fibres. Abundant glands with densely distributed, coarse granular, xanthophil secretion and numerous glands with amorphous, cyanophil secretion open through epithelial lining of penis papilla, besides sparse erythrophil glands through lining of penis papilla. Numerous glands with amorphous, cyanophil secretion and scattered glands with erythrophil, fine granular secretion open through epithelial lining of the atrium, which is cyanophil. Muscularis of penis papilla (40–80µm) composed of subepithelial layer with circular fibres followed by layer with longitudinal fibres, both layers well developed. Posteriorly to the gonopore, necks of cyanophil glands concentrate subepithelially; subepithelial muscle fibres of atrium scattered among these cell necks (Fig. 18).

Vitelline follicles, situated between intestinal branches, well developed. Ovaries ovoid, 1.5 times longer than wide, measuring 0.3 mm in its antero-posterior axis. They are located dorsally to the ventral nerve plate, in anterior sixth of body. Ovovitelline ducts emerge laterally from median third of ovaries, and run posteriorly immediately above nerve plate. Behind gonopore, the ovovitelline ducts ascend posteriorly and medially inclined, uniting to form a common glandular ovovitelline duct. This duct is situated dorsally to the relatively long, C shaped female canal, which opens into the atrium (Figs 15, 16).

Shell glands of two types: with coarse granular, erythrophil secretion, as well as with coarse granular, xanthophil secretion, the cells bodies of which occur among cell bodies of cyanophil glands. Towards female canal, the lining epithelium becomes pseudostratified and erythrophil. Two types of glands open through the epithelium of the female canal: erythrophil glands with finely granular secretion and cyanophil glands with amorphous secretion both sparsely distributed. Muscularis of female canal (20–40µm) composed of interwoven circular and longitudinal fibres.

Gonopore canal vertical at the sagittal plane. Common muscle coat highly developed, especially at penis bulb (Figs 15, 16), with interwoven oblique, circular and longitudinal fibres.

####### Etymology.

The name is a composite of the Latin adjective *cyano* (blue) and the Latin *atria*, referring to the abundant cyanophil secretion opening through the atrium.

####### Distribution.

Known only from the type-locality, Três Barras, Santa Catarina, Brazil.

## Notes on ecology and distribution

*Choeradoplanalongivesicula* was recorded only in its type-locality, the *Araucaria* Natural Heritage Private Reserve, state of Parana, in a site showing an initial stage of regeneration with poorly developed understorey (Rossi and Leal-Zanchet 2017, Amaral et al. 2018). *Choeradoplanacyanoatria* occurred only in its type-locality, the Três Barras National Forest, state of Santa Catarina, located ca. 150 km east from the type-locality of *C.longivesicula*, in an area of *Araucaria* moist forest. Both species showed low abundance during night samplings.

## Discussion

Both new species described herein match the diagnostic features of the genus *Choeradoplana*, namely a cephalic region that is curved backwards, a cephalic glandulo-muscular organ, and a cutaneous longitudinal musculature with a portion internal to the subcutaneous nerve plexus throughout the body, among others (Ogren and Kawakatsu 1990, Carbayo and Froehlich 2012, Carbayo et al. 2013).

Regarding external features, both new species resemble the type-species, *C.iheringi*, as well as *C.banga* Carbayo & Froehlich, 2012, *C.bocaina* Carbayo & Froehlich, 2012, *C.benyai* Lemos & Leal-Zanchet, 2014, *C.agua*Carbayo et al. 2017, *C.pucupucu*Carbayo et al. 2017 and *C.abaiba*Carbayo et al. 2017. All these species show a brownish dorsal pigmentation usually consisting of irregular, small dark-brown flecks (Carbayo and Froehlich 2012, Lemos et al. 2014, Carbayo et al. 2017).

With respect to the anatomy of the copulatory organs, by presenting a long, permanent penis papilla, the new species are easily differentiated from the species with an eversible penis papilla, namely *C.abaiba*, *C.agua*, *C.albonigra* (Riester 1938), *C.banga* Carbayo & Froehlich, 2012, *C.bocaina*, *C.gladismariae* Carbayo & Froehlich, 2012, *C.iheringi*, *C.langi* (Graff 1894), and *C.pucupucu* (Graff 1899, Riester 1938, Carbayo and Froehlich 2012, Carbayo et al. 2017). The presence of a long penis papilla horizontally disposed and occupying the whole length of the atrium distinguishes both new species from *C.bilix* Marcus, 1951, *C.catua* Froehlich, 1955, *C.marthae* Froehlich, 1955, and *C.crassiphalla* Negrete & Brusa, 2012. These four species have a short penis papilla that is obliquely disposed in the male atrium (Marcus 1951, Froehlich 1955, Negrete and Brusa 2012). *Choeradoplanalongivesicula* and *C.cyanoatria* also differ from *C.minima* Lemos and Leal-Zanchet, 2014, which shows an inverted penis (Lemos et al. 2014).

Both species share with *C.benyai* a long penis papilla with horizontal orientation. However, by having a cylindrical and long prostatic vesicle that traverses the penis papilla. *Choeradoplanalongivesicula* differs from *C.benyai* that shows a globose prostatic vesicle with folded wall restricted to the penis bulb (Lemos et al. 2014). In addition, a long ejaculatory duct traverses the penis papilla of *C.benyai*, whereas in *C.longivesicula* the prostatic vesicle opens into the tip of the penis papilla through a constriction, as if it were a short ejaculatory duct. Regarding shape of the prostatic vesicle and ejaculatory duct, *C.cyanoatria* is quite similar to *C.benyai* and can be easily differentiated from *C.longivesicula*. The elongate conical penis papilla of *C.cyanoatria* distinguishes it from *C.benyai*, which has a cylindrical and relatively longer penis papilla (Lemos et al. 2014). *Choeradoplanalongivesicula* and *C.cyanoatria* show a long atrium with a continuous muscle coat and without anatomical or histological distinction between male and female regions, thus differentiating them both from *C.benyai*. The latter shows male and female atria with independent muscle coats and different gland types opening into these regions (Lemos et al. 2014).

## Supplementary Material

XML Treatment for Choeradoplana longivesicula

XML Treatment for Choeradoplana cyanoatria
